# Primary Renal Carcinoid Tumor

**DOI:** 10.1100/tsw.2004.95

**Published:** 2004-06-03

**Authors:** Siamak Daneshmand, Shahin Chandrasoma, Shandra Wilson

**Affiliations:** Department of Urology, University of Southern California, Keck School of Medicine, Los Angeles, CA, USA

**Keywords:** kidney, carcinoid, renal

## Abstract

Primary carcinoid tumors originating in a normal kidney are exceptionally rare. We describe a case of a 52-year-old woman who presented with vague back pain and was found to have a 4-cm mass in the medial aspect of the left kidney. Radical nephrectomy and retroperitoneal lymphadenectomy were performed. Histopathology revealed a typical carcinoid tumor with 1 out of 17 lymph nodes involved. She has no evidence of recurrence or metastasis on 7-month follow-up.

## CASE PRESENTATION

A 52-year-old woman presented to her primary medical doctor with a 3-year history of lower back pain and right-sided posterior rib pain. A CT scan of the abdomen and pelvis revealed a 4-cm focally calcified mass arising from the medial aspect of the left kidney. The chest X-ray was normal. The patient was referred to urology for evaluation of a presumed left-sided renal cell carcinoma. There was no elevation of serum or urine catecholamines. She underwent a left radical nephrectomy with a retroperitoneal lymph node dissection. Grossly, there was a well-circumscribed, homogenously yellow mass measuring 3.2 cm in its greatest diameter projecting into the renal hilum (Fig. 1). The adrenal gland was normal. Histopathological sections of the mass showed a typical carcinoid tumor consisting of nests of small uniform cells with scanty cytoplasm and round nuclei that showed finely granular chromatin distribution (Fig. 2). Vascular invasion was identified, as was invasion into the renal capsule and perirenal fat; no invasion of Gerota’s fascia was seen. One out of 17 lymph nodes showed metastatic carcinoid tumor. Immunoperoxidase staining showed positivity for cytokeratin and synaptophysin, confirming the diagnosis of carcinoid tumor. The postoperative course was uneventful. Seven months after surgery, her serum 5-hydroxy indole acetic acid levels (5-HIAA) were normal and indium-111 octeotride scintigraphy was negative. She has no evidence of recurrence on CT or bone scan done at 6 months postoperatively.

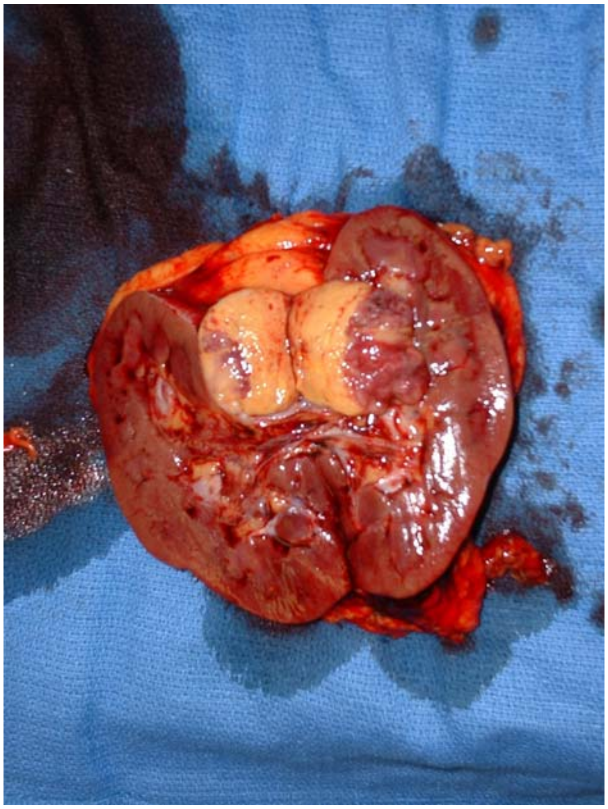

**FIGURE 1**. Photograph showing gross appearance of bisected kidney with hilar mass.

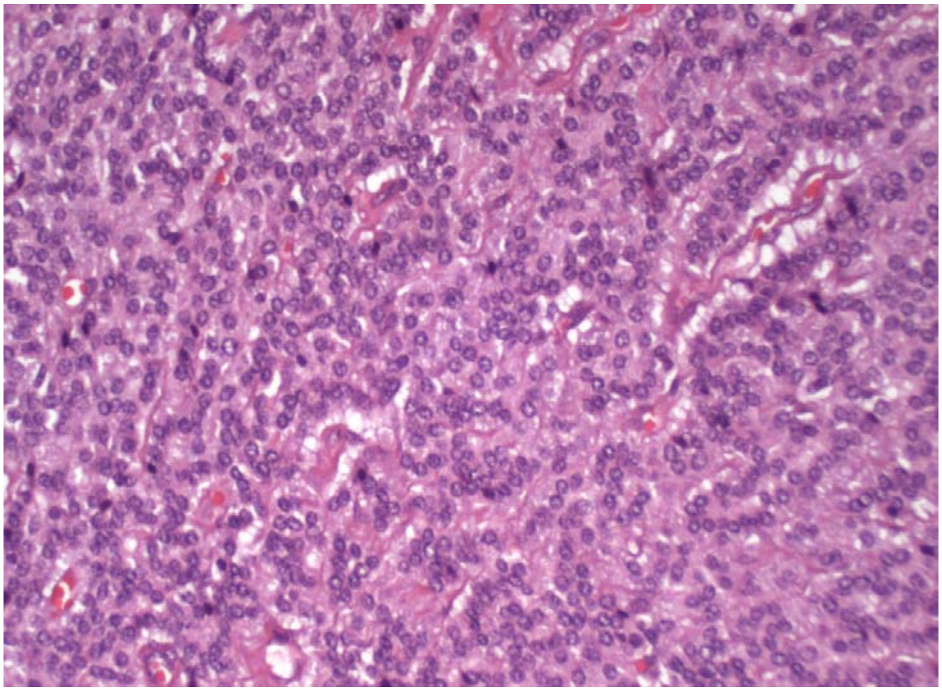

**FIGURE 2**. Microscopic section showing uniform small round cells in a trabecular pattern (Hematoxylin and eosin stain).

## DISCUSSION

Primary carcinoid tumors originating in a normal kidney are extremely rare entities. The pathogenesis of the primary renal carcinoid tumor is poorly understood because neuroendocrine cells are not found in the kidney. It is generally believed that the cell of origin for carcinoid tumor in the kidney is a pluripotential stem cell with capability for neuroendocrine differentiation. Some theorize that these cells may be of neural crest origin, trapped within the developing metanephros during embryological migration[1].

Renal carcinoid tumors have been reported with a relatively higher frequency in patients with abnormal kidneys. Of the 35 cases reported in the medical literature, 8 have arisen in horseshoe kidneys and 3 associated with renal teratomas. It has been suggested that these tumors arise from foci of hyperplastic neuroendocrine cells found in aberrant epithelium of the horseshoe kidney, which may represent intrarenal intestinal metaplasia or teratomatous degeneration of the renal parenchyma, making this pathogenetically equivalent to carcinoid tumors arising in renal teratomas[2,3].

Carcinoid tumors secrete 5-hydroxy tryptamine (serotonin), whose entry into the systemic circulation causes the carcinoid syndrome. The syndrome is characterized by diarrhea, flushing, asthma, and pulmonic valve stenosis, which may lead to right heart failure. In gastrointestinal tract carcinoid tumors, the carcinoid syndrome is almost always associated with metastasis because serotonin secreted by the primary tumor into the portal circulation is efficiently inactivated in the liver precluding it from reaching the systemic circulation. This is not true with carcinoid tumors of organs such as the bronchus, ovary, and kidney, where tumor secretion directly enters the systemic venous circulation. This patient had no evidence of carcinoid syndrome, most likely secondary to insufficient amount of hormones being released into the systemic circulation.

The patient is being followed with CT scans and serum 5-HIAA levels at 6-month intervals.
